# Pre-exposure prophylaxis with hydroxychloroquine for COVID-19: a double-blind, placebo-controlled randomized clinical trial

**DOI:** 10.1186/s13063-021-05758-9

**Published:** 2021-11-15

**Authors:** Berta Grau-Pujol, Daniel Camprubí-Ferrer, Helena Marti-Soler, Marc Fernández-Pardos, Clara Carreras-Abad, Maria Velasco-de Andrés, Elisabet Ferrer, Magdalena Muelas-Fernandez, Sophie Jullien, Giuseppe Barilaro, Sara Ajanovic, Isabel Vera, Laura Moreno, Eva Gonzalez-Redondo, Núria Cortes-Serra, Montserrat Roldán, Ana Artes-de Arcos, Isabel Mur, Pere Domingo, Felipe Garcia, Caterina Guinovart, Jose Muñoz

**Affiliations:** 1 Barcelona Institute for Global Health (ISGlobal), Hospital Clínic - University of Barcelona, Rosselló 132 4rt 1a, 08036 Barcelona, Spain; 2 Centro de Investigação em Saúde de Manhiça (CISM), Maputo, Mozambique; 3 Mundo Sano Foundation, Buenos Aires, Argentina; 4 Infectious Diseases Department, Hospital Clínic, IDIBAPS, University of Barcelona, Barcelona, Spain; 5 Infectious Diseases Unit, Hospital de la Santa Creu i Sant Pau - Institut d’Investigació Biomèdica Sant Pau, 08025 Barcelona, Spain; 6 Retrovirology and Viral Immunopathology, AIDS Research Group, IDIBAPS, Hospital Clinic, University of Barcelona, Barcelona, Spain

**Keywords:** Hydroxychloroquine, COVID-19, Pre-exposure prophylaxis, Prevention, Health-care workers, Control, SARS-CoV-2

## Abstract

**Background:**

Pre-exposure prophylaxis (PrEP) is a promising strategy to break COVID-19 transmission. Although hydroxychloroquine was evaluated for treatment and post-exposure prophylaxis, it is not evaluated for COVID-19 PrEP yet. The aim of this study was to evaluate the efficacy and safety of PrEP with hydroxychloroquine against placebo in healthcare workers at high risk of SARS-CoV-2 infection during an epidemic period.

**Methods:**

We conducted a double-blind placebo-controlled randomized clinical trial in three hospitals in Barcelona, Spain. From 350 adult healthcare workers screened, we included 269 participants with no active or past SARS-CoV-2 infection (determined by a negative nasopharyngeal SARS-CoV-2 PCR and a negative serology against SARS-CoV-2). Participants allocated in the intervention arm (PrEP) received 400 mg of hydroxychloroquine daily for the first four consecutive days and subsequently, 400 mg weekly during the study period. Participants in the control group followed the same treatment schedule with placebo tablets.

**Results:**

52.8% (142/269) of participants were in the hydroxychloroquine arm and 47.2% (127/269) in the placebo arm. Given the national epidemic incidence decay, only one participant in each group was diagnosed with COVID-19. The trial was stopped due to futility and our study design was deemed underpowered to evaluate any benefit regarding PrEP efficacy. Both groups showed a similar proportion of participants experiencing at least one adverse event (AE) (*p*=0.548). No serious AEs were reported. Almost all AEs (96.4%, 106/110) were mild. Only mild gastrointestinal symptoms were significantly higher in the hydroxychloroquine arm compared to the placebo arm (27.4% (39/142) vs 15.7% (20/127), *p*=0.041).

**Conclusions:**

Although the efficacy of PrEP with hydroxychloroquine for preventing COVID-19 could not be evaluated, our study showed that PrEP with hydroxychloroquine at low doses is safe.

**Trial registration:**

ClinicalTrials.govNCT04331834. Registered on April 2, 2020.

**Supplementary Information:**

The online version contains supplementary material available at 10.1186/s13063-021-05758-9.

## Background

The novel severe acute respiratory syndrome coronavirus 2 (SARS-CoV-2) is the causative agent of coronavirus disease 2019 (COVID-19) [1]. Currently, the worldwide prevention strategies for SARS-CoV-2 infection are vaccines, although self-protection equipment use, hand washing, case identification, case isolation, contact tracing, and exposed people quarantine of close contacts are still recommended [2–4]. However, vaccines were still under development at the time of the trial. In these circumstances, secondary attack rate estimates of COVID-19 ranged from 3 to 15% in the community [5–7], which can reach 26% in healthcare professionals [8]. Prevention of healthcare workers’ infection is crucial for protecting the workforce during pandemic management. Pre-exposure prophylaxis (PrEP) is a promising strategy, which proved effective in preventing other infectious diseases such as HIV [9]. Thus, several trials with repurposed drugs to evaluate PrEP effectiveness in mitigating SARS-CoV-2 transmission are under development [10–12].

Chloroquine was observed to effectively inhibit SARS-CoV-2 in vitro [13, 14]. Its derivate, hydroxychloroquine, shows a better in vitro antiviral activity and safety profile [15, 16]. Hydroxychloroquine potentially inhibits entry and post-entry stages of SARS-CoV-2 [13, 15]. While hydroxychloroquine was evaluated for the treatment of SARS-CoV-2 pneumonia and post-exposure prophylaxis, it is not evaluated for PrEP yet. The aim of this study is to compare the efficacy and safety of PrEP with hydroxychloroquine against placebo in healthcare workers in reducing the risk of COVID-19 disease during an epidemic period.

## Methods

### Trial design

We conducted a multicentre double-blind, placebo-controlled randomized clinical trial. We allocated participants to one of the two study arms in a 1:1 ratio by simple randomization. Randomization list was generated prior to enrolment. The trial protocol was described elsewhere [17].

Screening of candidates was initiated on April 3, 2020, and the first recruitment was on April 4, 2020.

This trial was approved by the Drug Research Ethics Committee of the Hospital Clinic of Barcelona (CEIm), Barcelona, Spain, and the Spanish Agency of Medicines and Medical Products (AEMPS). It was registered at clinicaltrials.gov (NCT04331834) on April 2, 2020.

### Participants

We recruited healthcare workers from three hospitals in Barcelona, Spain, (Hospital Clínic, Hospital de la Santa Creu i Sant Pau and Hospital Plató).

We included adult healthcare workers working at least 3 days a week in a trial hospital with a negative result of SARS-CoV-2 polymerase chain reaction (PCR) assay in nasopharyngeal swab within 4 days before enrolment. Serological testing to detect antibodies against SARS-CoV-2 was evaluated in all candidates with a rapid diagnostic test (Vivadiag^TM^ COVID-19 IgM/IgG Rapid Test©, Hangzhou, China) and confirmed with Enzyme-Linked ImmunoSorbent Assays (VITROS Anti-SARS-CoV-2 Total© Ortho-Clinical Diagnostics, 2020). Those individuals with a positive COVID-19 serological testing by any method were excluded. Participants with any of the following conditions were also excluded: pregnancy, breastfeeding, ongoing antiviral, antiretroviral or corticosteroids treatment, chloroquine or hydroxychloroquine intake the last month, or any contraindication to hydroxychloroquine.

### Intervention and comparator

Randomization was generated using a computer random number generator. We used sequentially numbered sealed envelopes of identical appearance containing either hydroxychloroquine or placebo, ensuring allocation concealment. Participants allocated to the intervention arm (PrEP) received 400 mg of hydroxychloroquine (two tablets of 200 mg) daily the first four consecutive days, followed by 400 mg weekly during the study period, initially scheduled to be 6 months. Participants in the control group followed the same treatment schedule with placebo tablets that were indistinguishable from hydroxychloroquine tablets.

Participants took the first two tablets at the recruitment visit under direct observation by a physician, who then provided the needed tablets to complete the first month of treatment.

Participants, investigators assessing participant eligibility and recruitment, assessing outcomes and follow-up, and/or dealing with data management and analysis were all blinded to arm allocation. Only one person unrelated to participant recruitment and follow-up, clinical assistance, data management, and analysis had access to this information.

### Outcome

The primary outcome was the incidence of COVID-19 confirmed cases (defined by compatible symptoms with COVID-19 with seroconversion or a positive PCR for SARS-CoV-2) in the hydroxychloroquine arm compared to the placebo arm at any time during the study follow-up.

The secondary outcomes included (i) the SARS-CoV-2 seroconversion in the hydroxychloroquine group compared to the placebo group in both asymptomatic and symptomatic participants; (ii) the occurrence of any adverse event (AE) related with hydroxychloroquine treatment; (iii) the incidence of SARS-CoV-2 infection and COVID-19 in healthcare workers in the placebo group during the study period; and (iv) the risk ratio for the different clinical, analytical and microbiological conditions to develop COVID-19.

### Participant’s follow-up

Passive and active surveillance was conducted on all participants to detect SARS-CoV-2 infections and any AE.

Active surveillance of each participant was conducted monthly by blinded physicians unaware of the trial arm assignments, which completed a standardized case report form (CRF) for each participant. Follow-up visits included (i) assessment of compliance with PrEP; (ii) physical examination and detailed evaluation of symptoms to either detect past and current symptoms and signs related to COVID-19, as well as possible adverse events; (iii) venepuncture for blood determinations, including SARS-CoV-2 serology test (VITROS Anti-SARS-CoV-2 Total© Ortho-Clinical Diagnostics, 2020); (iv) assessment of COVID-19 risk factors such as known close contacts with suspected and/or confirmed COVID-19 cases or the number of weeks during which they were managing COVID-19 patients; (v) standardized questions to collect past and current common side effects along with open free text; and (vi) electrocardiogram to evaluate possible cardiac rhythm alterations.

During this study period, a medical doctor was available by phone 24 h a day for passive surveillance. All participants were provided with this contact number in case of presenting any COVID-19 related symptom or AE. In that instance, a standardized CRF was filled out to collect the information. A nasopharyngeal swab was performed on all those participants presenting with COVID-19-related symptoms to detect SARS-CoV-2 infection by PCR. Adverse events were thus assessed during the participant’s follow-up visits with a standardized CRF for each participant, performing an electrocardiogram to evaluate possible cardiac rhythm alterations and if they contacted the provided phone number for assistance. AEs were recorded in a specific adverse event reporting form and were measured based on the ICH-GCP guidelines, determining the severity of the event, the relationship to study intervention, and expectedness of the adverse event. All these classifications were performed by blinded physicians unaware of the trial arm assignments.

Medical assistance was ensured for all participants diagnosed with SARS-CoV-2 infection following hospital guidelines.

Although the protocol was designed to follow participants for 6 months, this manuscript only includes participants’ first-month analysis.

### Sample size

We estimated sample size assuming an expected incidence of 10% of COVID-19 in healthcare workers in the control group and 2% in the hydroxychloroquine group, with a hazard ratio of 0.2. Thus, we required a total of 440 subjects (220 per group) for a significance level of 5%, statistical power of 90%, and assuming a rate of lost-to-follow-up of 10% [18].

### Interim analysis

Interim analyses of the efficacy and safety of hydroxychloroquine were planned monthly, with the option of early stopping the trial for futility. We planned to re-estimate incidence and lost to follow-up rate at the first month, since these data were unknown when we estimated sample size. After the first interim analysis, the trial was halted on the basis of a very low incidence rate among study participants. Thus, results were only provided for the first month.

### Statistical analysis

We conducted an intention-to-treat analysis, with all patients fulfilling inclusion criteria and not presenting exclusion criteria. Categorical variables were expressed as absolute frequency and percentage and were compared with Fisher’s exact test. Continuous variables were expressed as mean and standard deviation (SD) or median and interquartile range (IQR). We conducted all analyses with R [19].

## Results

### Participants

#### Screening

**Fig. 1:**
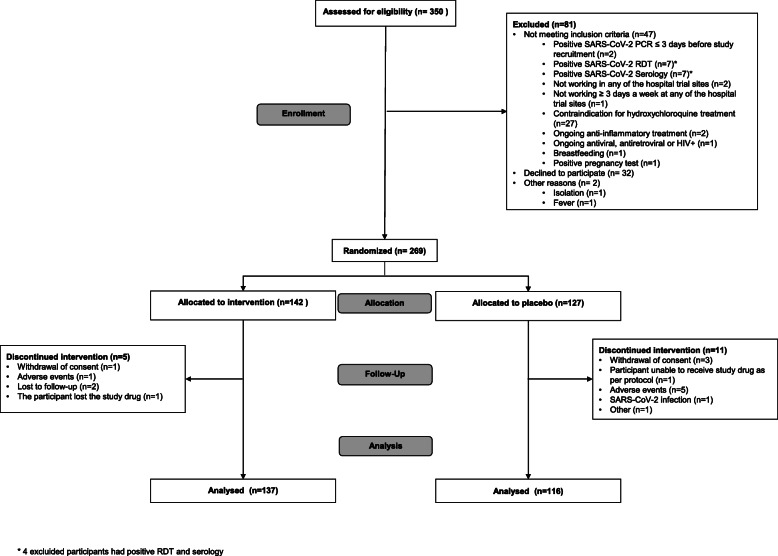
Flow diagram of trial participants at screening, recruitment, and follow-up

We assessed 350 healthcare workers for eligibility; 269 of them fulfilled the study criteria and were recruited after signing the informed consent form. Participants were randomly assigned to the hydroxychloroquine group (*n*= 142, 52.8%) and to the placebo group (*n*= 127, 47.2%). The trial had to be stopped due to the low recruitment rate, and the estimated sample size was not reached. Figure 1 describes participants’ enrolment and randomization.

The participants’ demographic characteristics are shown in Table 1. The trial included 197/269 (73.2%) female participants and the median age was 39 years [IQR: 30–50]. Eighty-three (30.9%) had some underlying medical condition and 74 (25.5%) were under chronic treatment.

**Table 1:** Participants’ baseline characteristics for both the hydroxychloroquine and placebo group

	Placebo (*n* = 127)		Hydroxychloroquine (*n* = 142)	
	*n*	%	*n*	%
Sex, female	93	73.2	104	73.2
Age (mean, SD)	40.3	(12.8)	39.6	(11.2)
Country of origin				
Spain	112	88.2	126	88.7
Other European countries	0		4	2.8
Latin America	14	11.0	12	8.5
North Africa	1	0.8	0	
Professional category				
Medical Doctor	53	42.1	67	47.2
Nurse	35	27.8	40	28.2
Nurse Assistant	12	9.5	12	8.5
Administrative	10	7.9	10	7.0
Other	16	12.7	13	9.2
Smoking	17	13.8	21	14.9
**Comorbidities**				
Any	42	33.1	41	28.9
Diabetes mellitus	1	0.8	0	
Hypertension	3	2.4	2	1.4
Chronic respiratory condition	2	1.6	5	3.5
Dyslipidemia	4	3.1	3	2.1
Hyperthyroidism	6	4.7	7	4.7
Allergy	3	2.4	2	1.4
Dermatological condition	5	3.9	2	1.4
Gastrointestinal/liver conditions	2	1.6	5	3.5
Gynecological conditions	2	1.6	4	2.8
Psychological disease	2	1.6	2	1.4
Neurological disease	1	0.8	3	2.1
Other	16	12.6	8	5.6
**Immunosuppression**	0		0	
**Chronic treatment**				
Any	34	26.8	40	28.2
Antidiabetics	0		1	0.7
Antihypertensives	1	0.8	2	1.4
Statins	3	2.4	1	0.7
Bronchodilators	2	1.6	1	0.7
Contraceptives	9	7.1	11	7.7
Levothyroxine	6	4.7	9	6.3
Proton pump inhibitors	2	1.6	5	3.5
Other	5	3.9	4	2.8
**History of vaccination**				
Haemophilus	9	7.8	8	6.2
Pneumococcal	5	4.2	9	6.7
Influenza	85	68.5	92	64.8

*SD* standard deviation

Almost half of the recruited individuals (44.6%, 120/269) were medical doctors. 33.1% referred contact with at least one confirmed or probable COVID-19 case without wearing a mask, 34.6% (44/127) in the hydroxychloroquine, and 30.2% (43/142) in the placebo group (*p* value=0.526). From them, the median number of contacts per participant was 2.5 [IQR: 2–3]. Participants were living with a median number of 2 co-habitants [IQR: 1–3] and 28.3% (76/269) of the participants were living with healthcare workers. All studied risk factors were similar between groups. Table 2 describes participants’ risk factors for COVID-19 exposure.

**Table 2:** Participant’s risk factors of COVID-19 exposure at screening and the first month of follow-up

	Screening				Month 1			
	Placebo (*n*=127)		Hydroxychloroquine (*n*=142)		Placebo (*n*=116)		Hydroxychloroquine (*n*=137)	
**Number of cohabitants** (median, IQR)	2	[1–3]	1.5	[1–3]	1	[1–3]	2	[1–3]
Confirmed cases (median, IQR)	1	[1–1]	1	[1–1]	1	[1–1]	1	[1–1]
Suspected cases (median, IQR)	3	[3–3]	3	[3–3]	3	[3–3]	3	[3–3]
**Used public transportation**^**a**^	54	43.2	55	38.7	56	48.7	58	42.3
**Close contact with animals**^**a**^	26	20.8	36	25.5	39	33.6	52	38.0
**Use of COVID-19 recommended PPE at work**^**a,b**^								
Always	107	86.3	117	82.4	111	95.7	135	98.5
Almost always	8	6.5	11	7.7	4	3.4	2	1.5
Sometimes	2	1.6	5	3.5	1	0.9	0	0.0
Occasionally	2	1.6	1	0.7	0	0.0	0	0.0
Never	5	4.0	8	5.6	0	0.0	0	0.0
**Close contact with a confirmed COVID-19 case without using PPE**^**a**^	35	28.0	34	23.9	17	14.7	7	5.1
If yes, how many								
1	20	57.1	11	32.4	9	56.2	3	42.9
2–3	10	28.6	18	59.2	7	43.8	3	42.9
≥ 4	5	14.3	5	14.7	0	0	1	14.3
If yes, how frequently								
Every day	16	48.5	13	38.2	2	11.8	0	0.0
≥ 1/week	8	24.2	10	29.4	8	47.1	5	83.3
< 1/week	9	27.3	13	38.2	9	42.1	1	16.7
**Close contact with a suspected COVID-19 case without using PPE**^**a**^	19	15.2	15	10.6	5	4.3	6	4.4
If yes, how many								
1	7	36.8	6	40.0	1	20.0	3	50.0
2–3	6	31.6	7	46.7	2	40.0	2	33.3
≥ 4	6	31.6	2	13.3	2	40.0	1	16.7
If yes, how frequently								
Every day	8	44.4	3	20.0	1	20.0	1	16.7
≥ 1/week	6	33.3	8	53.3	2	40.0	4	67.7
< 1/week	4	22.2	4	26.7	2	40.0	1	16.7

*Sometimes* defined as not using the proper PPE 1-2 times/week. *Occasionally* defined as not using the proper PPE > 2 times/week

*PPE* personal protection equipment

^a^During the last 20 days

^b^*Almost always* defined as not using the proper PPE protection 1–2 times in the last 20 days

Included participants did not present any relevant abnormality in the blood test (Table 3) neither in the electrocardiogram. Median QTc did not differ between groups (383 ms [IQR: 357–400] in the hydroxychloroquine group and 384 ms [IQR: 368–405] in the placebo group).

**Table 3:** Comparison of adverse events (AE) between study groups after 1 month of follow-up

	Placebo (*n* = 127)		Hydroxychloroquine (*n*=142)		
	*n*	%	*n*	%	*p* value
**At least 1 adverse event**	42	33.1	53	37.3	0.548
**Syndromic approach**					
**General symptoms**	9	7.7	10	7.0	> 0.999
Fever	6	4.7	4	2.8	0.553
Chills	0	0.0	2	1.4	0.552
Sweating	0	0.0	0	0.0	
Malaise	4	3.1	4	2.8	> 0.999
Myalgia	2	1.6	3	2.1	> 0.999
Arthralgia	0	0.0	0	0.0	
**Gastrointestinal symptoms**	20	15.7	39	27.4	0.041
Nausea	3	2.4	10	7.0	0.160
Abdominal pain	11	8.3	15	10.9	0.825
Diarrhea	8	6.3	24	16.9	0.028
Dysgeusia	0	0.0	0	0.0	
**Dermatological symptoms**	2	1.6	3	2.2	> 0.999
Itching	0	0.0	2	1.4	0.546
Rash	2	1.6	2	1.4	> 0.999
**Respiratory symptoms**	9	7.1	5	3.7	0.257
Rhinorrea	3	2.4	0	0.0	0.190
Sore throat/odynophagia	5	3.9	3	2.2	0.556
Cough	3	2.4	2	1.5	0.851
Pleuritic pain	0	0.0	0	0.0	
Dyspnea	0	0.0	0	0.0	
**Neurological symptoms**	12	9.4	14	9.9	> 0.999
Headache	12	9.4	13	9.1	0.987
Visual disturbances	0	0.0	1	0.7	>0.999
**Cardiovascular symptoms**	2	1.6	2	1.4	0.999
**Other symptoms**	10	8.0	7	4.9	0.427
**Severity**					0.249
Mild	43	33.8	63	44.4	
Moderate	3	2.4	1	0.7	
Severe	0	0.0	0	0.0	
**Potential relationship with the study drug**					
Related (at least one AE)	33	26.0	49	34.5	0.206
Non related (at least one AE)	17	13.4	14	9.9	0.476
**Withdrawal due to AE**	5	3.9	1	0.7	0.270

% according to available data

#### Withdrawal and lost to follow-up

On June 12th, a total of 253 (94.1%) participants had completed the first month of follow-up. In the hydroxychloroquine group, 137 (96.5%) completed follow-up at first month. The reasons for no completion were withdrawal of consent (*n*=1, 0.7%), AEs (*n*=1, 0.7%), and others (*n*=3, 2.1%). In the placebo group, 116 (91.3%) completed the course of prophylaxis. Their reasons of no completion were withdrawal of consent (*n*=3, 2.4%), participant unable to receive study drug as per protocol (*n*=1, 0.8%), AEs (*n*=5, 3.9%), SARS-CoV-2 infection (*n*=1, 0.8%), and others (*n*=1, 0.8%) (Fig. 1).

#### COVID-19 risk factors at month 1

After a month of follow-up, 39.4% (65/165) of participants ceased to work in a COVID-19 hospital unit, with a similar proportion between both groups. In addition, only seven participants denied having always used PPE when assisting patients. The proportion of participants in contact with a confirmed and/or a suspected COVID-19 case showed to be lower compared with the screening visit (*p* value< 0.001 in the hydroxychloroquine group, *p* value=0.002 in the placebo group). Participants from the hydroxychloroquine group had lower contact with confirmed COVID-19 cases without using PPE compared to the placebo (5.1% (7/137) vs 14.7% (17/116), respectively, *p* value=0.018) (Table 2).

### Efficacy

During the first month of follow-up, the cumulative incidence of COVID-19 among the study participants was 0.4%. Among all trial participants at the end of the first month (*n* = 253), only one participant from the placebo arm (1/116, 0.8%), tested positive forSARS-CoV-2 PCR and for a SARS-CoV-2 serology test. The participant presented with fever, respiratory symptoms, and headache 6 days after randomization. The participant did not receive specific treatment for COVID-19 or required hospitalization.

The risk ratio of collected risk factors and clinical and analytical conditions for developing COVID-19 could not be calculated due to low COVID-19 incidence.

### Safety

A total of 95 participants experienced at least one AE during the first month of follow-up, posing an overall accumulated prevalence of 35.3%. The proportion of participants experiencing at least one AE was similar in both groups. Eighty-two events (34.5% (49/142) in the hydroxychloroquine group and 26.0% (33/127) in the placebo group, *p*=0.206) were judged to be related to the study intervention (hydroxychloroquine or placebo). No serious AEs were reported. Almost all AEs (96.4%, 106/110) were considered mild. Only four were reported as moderate: prostate adenocarcinoma in the hydroxychloroquine group and dental infection, hypertensive crisis, and myalgia in the placebo group.

Gastrointestinal symptoms (diarrhea, abdominal pain, and nausea) were the most common AEs, and they were more commonly reported in the hydroxychloroquine group. The median number of days from the first dose to intake to AE appearance was 2.5 in the hydroxychloroquine group [IQR 0–10.5] and 6 in the placebo group [IQR 2–18].

Headache, rash, respiratory, and general symptoms were also observed. They appeared similarly in time between both groups; the median number of days to appearance was 4.5 [IQR: 0–17] in the hydroxychloroquine group and 5 [IQR: 1–18] in the placebo group.

Four cardiovascular AEs (two in each study group) were detected during the first month of treatment: two pre-excitation syndromes (Wolf-Parkinson-White), one with hypertensive crisis, and two participants presenting with heart palpitations. The only moderate cardiovascular AE was the hypertensive crisis; the rest were mild and none of them was considered related to the study drug. None of the participants presented prolonged QTc intervals at first month (388 ms [IQR: 365–402] in the hydroxychloroquine group and 393 ms [IQR: 371–405] in the placebo group).

Only one participant in the hydroxychloroquine group presented mild visual disturbances.

One participant discontinued the prophylaxis due to AEs in the hydroxychloroquine group and five in the placebo group. Table 3 shows a detailed description of the AEs presented in both study groups.

No relevant laboratory abnormalities occurred. Most abnormalities were transient, with no significant changes in the two groups (Supplementary Table 1).

## Discussion

Although the SARS-CoV-2 incidence found in study participants was low, seroprevalence studies show that healthcare workers are at high risk of being infected by SARS-CoV-2 [20]. Thus, major efforts should be made to protect these essential workers from the infection, primarily by prophylactic measures (chemoprophylaxis or vaccination). We conducted a multicentre double-blind placebo-controlled randomized clinical trial to evaluate whether PrEP with hydroxychloroquine was an effective intervention for preventing COVID-19 among healthcare workers during a COVID-19 epidemic period. These data showed that prophylaxis with hydroxychloroquine at the study doses had an excellent safety profile. Nevertheless, the community incidence of SARS-CoV-2 events decreased during the first month of follow-up as a consequence of the country’s control and mitigation strategies. Thus, the overall incidence in the cohort was 0.8%, the trial was stopped due to futility, and the study design was deemed underpowered to answer the main objective. Although the SARS-CoV-2 incidence found in study participants was low, seroprevalence studies show that healthcare workers are at high risk of being infected by SARS-CoV2 [20]. Thus, major efforts should be made to protect these essential workers from the infection, primarily by prophylactic measures (chemoprophylaxis or vaccination). These strategies should also be evaluated in other populations that are likely to be at high risk of exposure, such as patients and staff at long-term care facilities or people in other congregate living situations.

In this trial, AEs were similar between the hydroxychloroquine and the placebo group. Mild gastrointestinal events were higher in the hydroxychloroquine group than in the placebo group. Our safety data contrast with available data from a recently published placebo-controlled clinical trial post-exposure prophylaxis with hydroxychloroquine to prevent COVID-19, in which the intervention arm had a higher rate of AEs (40.1%) compared to the placebo arm (16.8%) [21]. The reasons for these discrepancies may include the high loading doses of hydroxychloroquine required for the post-exposure prophylaxis strategies (3200 mg compared to 1200 mg in 4 days in our intervention). Moreover, the loading dose during 4 consecutive days in PrEP strategies could be avoided since the patient has not yet been exposed to the virus, potentially decreasing the number of AEs.

Large observational studies evaluating the hydroxychloroquine effect on SARS-CoV-2 pneumonia showed that higher doses of the drug could be associated with QTc interval prolongation and death due to cardiovascular events; especially when administered with other drugs which favor QT interval prolongation [22, 23]. The relationship of high doses of hydroxychloroquine with severe AEs was also supported by a recent study evaluating high doses of hydroxychloroquine (up to 600 bid during 10 days) for treating COVID-19 patients. This study showed that 15% of participants had prolonged QTc interval and two of them presented ventricular tachycardia. In this specific case, the authors suggested that this outcome could have been influenced by most of their participants receiving oseltamivir, which also prolongs QT, and the older age of them [24]. In our case, no cardiovascular events related to the study drug were observed.

The adequate safety profile of the preventive (low) doses of hydroxychloroquine found in our study is supported by many studies on hydroxychloroquine short-term use as antimalarial and long-term use for rheumatic diseases demonstrated to be safe as well [25–28].

As mentioned above, this trial has some limitations. The main one is a low power in our study design to assess the efficacy of PrEP with hydroxychloroquine in healthcare workers at the first interim analysis. The principal reason was the low incidence of COVID-19 during the study follow-up in the study area. The analysis presented in this manuscript was conducted during April, May, and June 2020 in Barcelona. During that period, COVID-19 reported cases were already decreasing in Catalunya: while 1208 cases were reported on April 3rd, 77 cases were reported on June 12th. Accordingly, SARS-CoV-2 attack rate declined from 0.9 at the initiation of the study to 0.77 at the end of the analysis [29]. In addition, we noticed that healthcare workers' risk perception decreased as the national epidemic vanished. That had an impact not only on participants’ recruitment but also on participants’ adherence and follow-up.

Moreover, other factors influenced their participation. Some study candidates had already self-prescribed hydroxychloroquine assuming its role on COVID-19 prevention, so although displaying interest, they were excluded to participate. In addition, a fraction of recruited participants dropped out from the study the following day of test results notification. Hence, this set of circumstances prompted us to stop trial recruitment by the 8th of May even though we had not reached our estimated sample size. Consequently, both study groups were unevenly distributed (52.8% vs 47.2%).

## Conclusions

This trial displayed that administering 400 mg of hydroxychloroquine during 4 consecutive days followed by 400 mg of hydroxychloroquine weekly in adults during a month was safe. The question if COVID-19 could be prevented with hydroxychloroquine PrEP was unanswered. Since the epidemiological situation happening in our country is expected to be reproduced in other areas where similar trials are being conducted, our group will make all efforts to share databases with clinical trials with a similar design, doses of hydroxychloroquine, and similar study endpoints, in an effort to answer the main question that initially fostered the design of this and similar studies. Preventing healthcare workers from contracting COVID-19 is critical to control the pandemic. Thus, further studies in countries in the peak of the pandemic are needed to investigate effective preventive measures.

## Supplementary Information

**Additional file 1.**

## Data Availability

Data will be available from the author on reasonable request (jose.munoz@isglobal.org).

## Abbreviations

AE: Adverse event
AEMPS: Spain and the Spanish Agency of Medicines and Medical Products
CEIm: Drug Research Ethics Committee of the Hospital Clinic of Barcelona
CRF: Case report form
CT: Cycle threshold
IQR: Interquartile range
PCR: Polymerase chain reaction
PrEP: Pre-exposure prophylaxis
SARS-CoV-2: Severe acute respiratory syndrome coronavirus 2
SD: Standard deviation
WHO: World Health Organization
