# Supplementary material for: Pre-exposure prophylaxis with hydroxychloroquine for COVID-19: a double-blind, placebo-controlled randomized clinical trial
Source: Trials. 2021 Nov 15;22:808. doi: 10.1186/s13063-021-05758-9 (PMC8591593; doi:10.1186/s13063-021-05758-9)
Supplement: Supplementary file 1 — Additional file 1. [file 13063_2021_5758_MOESM1_ESM.docx]

**Supplementary Table 1 – Laboratory parameters at screening and first month of follow-up.**

|  | Reference  values | Screening | | | | Month 1 | | | | | |
| --- | --- | --- | --- | --- | --- | --- | --- | --- | --- | --- | --- |
|  |  | Placebo | | Hydroxychloroquine | | Placebo | | | Hydroxychloroquine | | |
|  |  | Median | IQR | Median | IQR | Median | IQR | | Median | Q1 - Q3 | |
| WBC | 4.0 – 11.0 x10^9^/L | 6.31 | (5.38 - 7.24) | 6.13 | (5.16 - 7.18) | 5.88 | (4.99 - 6.98) | | 5.92 | (5.11 - 6.70) | |
| Neutrophils | 2.0 – 7.0 x10^9^/L | 3.6 | (2.90 - 4.46) | 3.5 | (2.95 - 4.44) | 3.4 | (2.8 - 4.4) | | 3.49 | (2.6 - 4.2) | |
| Lymphocytes | 0.9 – 4.5 x10^9^/L | 2.0 | (1.6 - 2.3) | 1.9 | (1.6 - 2.2) | 1.8 | (1.4 - 2.2) | | 1.8 | (1.6 - 2.1) | |
| Eosinophils | <0.5 x10^9^/L | 0.1 | (0.1 - 0.2) | 0.1 | (0.1 - 0.2) | 0.1 | (0.1 - 0.2) | | 0.2 | (0.1 - 0.3) | |
| Basophils | <0.2 x10^9^/L | 0 | (0 - 0) | 0 | (0 – 0) | 0 | (0 – 0) | | 0 | (0 – 0) | |
| Monocytes | 0.1 – 1.0 x10^9^/L | 0.4 | (0.3 - 0.4) | 0.3 | (0.3 - 0.4) | 0.3 | (0.3 - 0.4) | | 0.3 | (0.3 - 0.4) | |
| Platelets | 130 – 400 x10^9^/L | 239 | (215 - 266) | 235 | (198 – 265) | 237 | (210 – 270) | | 223.5 | (198.3 - 264.5) | |
| Haemoglobin | 130 – 170 g/L | 137.5 | (129 - 147.3) | 138 | (131 - 147) | 136 | (129 – 143) | | 138 | (131 – 147) | |
| Haematocrit | 0.40 – 0.50 L/L | 0.43 | (0.40 - 0.46) | 0.43 | (0.39 - 0.45) | 0.41 | (0.39 - 0.45) | | 0.42 | (0.40 - 0.44) | |
| CRP | <0.40 mg/dL | <0.40 | (<0.40 - <0.40) | <0.40 | (<0.40 - <0.40) | <0.40 | (<0.40 - <0.40) | | <0.40 | (<0.40 - <0.40) | |
| ASAT | 5 – 40 IU/L | 21.0 | (18 – 26) | 20 | (17 – 23) | 21 | (18 – 25) | | 20 | (17 – 23) | |
| ALAT | 5 – 40 IU/L | 17 | (13.8 – 26) | 16 | (13 – 20) | 17 | (13 – 22) | | 15 | (12 – 20) | |
| GGT | 5 – 40 IU/L | 18 | (14 – 23) | 16 | (13 – 23) | 17 | (13 – 21) | | 16 | (12 – 21) | |
| AP | 46 – 116 IU/L | 63.5 | (52 - 77.8) | 57 | (50 – 72) | 65 | (51 – 77) | | 54 | (47 – 68) | |
| Bilirubin | < 1.2 mg/dL | 0.59 | (0.4 - 0.7) | 0.6 | (0.5 - 0.8) | 0.6 | (0.5 - 0.8) | | 0.6 | (0.5 - 0.8) | |
| Creatinine | 0.30 – 1.30 mg/dL | 0.76 | (0.69 - 0.85) | 0.75 | (0.66 - 0.86) | 0.78 | (0.71 - 0.88) |  | 0.75 | (0.69 - 0.88) |  |
| Na | 135 – 145 mEq/L | 140 | (140 – 141) | 141 | (140 – 142) | 140 | (138 – 141) | | 139 | (138 – 141) | |
| Glucose | 65 – 110 mg/dL | 82 | (75 - 87.92) | 82 | (77 - 88.75) | 86.76 | (78 – 96) | | 87.00 | (80.25 - 94.75) | |
| D-dimer | < 500 ng/mL | 200 | (200 – 300) | 200 | (200 – 300) | 200 | (200 – 300) | | 200 | (200 - 300) | |

ALAT: alanine aminotransferase. AP: alkaline phosphatase. ASAT: aspartate aminotransferase. CRP: C-reactive protein. GGT: Gamma-glutamyl transpeptidase. IQR: Interquartile range. Na: Sodium. WBC: white blood cells.
